# The role of HIF-1α in innate immune mechanisms and autoimmunity: A double-edged sword

**DOI:** 10.1016/j.jbc.2026.111429

**Published:** 2026-05-11

**Authors:** Martha L. Chapple, Arnaud Zaldumbide, Raphael R. Fagundes

**Affiliations:** Department of Cell & Chemical Biology, Leiden University Medical Center (LUMC), Leiden, The Netherlands

**Keywords:** autoimmunity, HIF-1, hypoxia, innate immunity, type 1 interferon

## Abstract

Millions of people all around the world are affected by autoimmune diseases, characterized by immune-mediated destruction of cells or tissues and consequent organ dysfunction. Recently, the role of innate immunity in autoimmune disease pathogenesis has been established, with an emphasis on the type 1 interferon response. Based on findings from cancer research, the role of the hypoxic tumor microenvironment in suppressing the type 1 interferon response and subsequent immune activation has steered autoimmunity research toward the immunomodulatory effects of hypoxia-induced activation of the hypoxia-inducible factor-1α (HIF-1α) pathway. Although the immunostimulatory effects of HIF-1α in certain autoimmune diseases are well documented, its role and underlying mechanisms remain controversial. Here, we present an overview of HIF-1α-mediated immunomodulation and gain insight into the mechanisms behind the contradictory effects. We will discuss mechanisms through which disruptions in innate immunity contribute to autoimmune disease pathogenesis and the divergent immunomodulatory effects that HIF-1α exerts in various autoimmune disorders. Finally, we will examine the pharmacological potential of HIF-1α modulation to regulate innate immunity prior to the development of autoimmune diseases.

Autoimmune diseases encompass almost 100 disorders characterized by inflammation and organ dysfunction because of immune attack against host cells or tissues. Taken together, 3% to 5% of the global population is affected by any autoimmune disease, with an inexplicable increased frequency among women, resulting in a tremendous economic, social, and quality-of-life burden (1, 2). The wide range of autoimmune diseases includes more prevalent disorders, such as systemic lupus erythematosus (SLE) and type 1 diabetes mellitus (T1DM), as well as rarer forms like ankylosing spondylitis. Although clinical manifestations vary from organ specific to systemic, all autoimmune disorders are characterized by immune dysregulation and are commonly diagnosed based on the presence of autoantibodies or autoreactive T cells (3).

While the adaptive immune system is ultimately responsible for autoimmune destruction, the exact mechanisms driving autoimmunity remain elusive. For instance, genetic predisposition is a well-established factor, with certain autoimmune diseases arising from single-gene mutations, including the rare IPEX (immune dysregulation, polyendocrinopathy, enteropathy, X-linked) syndrome, caused by mutations in *FOXP3*, and autoimmune polyendocrine syndrome type 1, which results from an absence of the transcription factor autoimmune regulator, among others (3, 4, 5, 6). However, most disorders require additional environmental triggers, often including previous exposure to infectious agents (1). This is hypothesized to cause an autoimmune attack by recognition of self-antigens resembling pathogenic antigens. Although such molecular mimicry has not been proven in most autoimmune conditions, this hypothesis is supported by the presence of circulating innate type 1 interferons (T1-IFNs) prior to disease onset (7, 8, 9, 10).

T1-IFNs mediate innate antiviral immune response by containing infected cells through interference at various phases of viral replication and priming adjacent, uninfected cells for an eventual infection by inducing the transcription of interferon-stimulated genes (ISGs) and secretion of IFN-⍺ and IFN-β (11, 12, 13). This, in turn, enhances immune cell cytotoxicity and boosts inflammation (11, 12). While T1-IFNs are predominantly involved in antiviral immunity, detection before autoimmune disease onset indicates an important role of the innate immune system in autoimmunity (7, 14, 15, 16). Aberrant activation of T1-IFNs and additional components of innate immunity have been implicated in the development of autoimmune disorders, including, but not limited to, SLE and T1DM (17, 18, 19). However, the exact connection between innate immunity and autoimmunity remains unclear, and understanding this is essential to uncover potential targets in the treatment or prevention of autoimmune diseases.

Extensive evidence points to a role of the microenvironment, such as physiological gases, extracellular matrix components, infections, and cytokines, in modulating both immunosuppressive and immunostimulatory phenotypes (12, 20). Low oxygen tension in affected tissues (*i.e.*, microenvironmental hypoxia) is a key cue implicated in immunomodulation, with cancer research highlighting its importance in suppressing innate immune responses, thereby contributing to immune evasion (12, 21). Conversely, hypoxia is a common feature of many complications of autoimmune disorders, suggesting an additional immunostimulatory role of hypoxia (22, 23). Despite evidence of both immunosuppressive and immunostimulatory effects of hypoxia, the mechanisms that determine whether oxygen-sensitive pathways exert a protective or a detrimental role across autoimmune diseases remain poorly understood (24, 25, 26).

This review seeks to explore the mechanisms through which hypoxia-inducible factor-1α (HIF-1α), the key transcriptional factor regulating the intracellular response to hypoxia, acts as a double-edged sword by influencing innate immune mechanisms to either prevent or promote autoimmune responses. We will compare studies that investigate how disruptions in intracellular signaling trigger the innate immune system to initiate autoimmune responses and analyze evidence aimed at identifying the innate immunomodulatory mechanisms of HIF-1α activation. Furthermore, we will examine the processes driving the divergent functions of HIF-1α on mechanisms of innate immunity, where it can either promote immune activation and autoimmunity or suppress immune responses to prevent autoimmunity. Finally, we will discuss potential strategies for modulating HIF-1α in the context of autoimmunity. Since such interventions are ineffective once an autoimmune disease has developed, it is crucial to unravel pathophysiological phenotypes to target mechanisms that could help prevent the onset of autoimmune diseases.

## Innate immune mechanisms and autoimmunity

Innate immunity represents the first line of defense and is present in all nucleated cells of the human body, comprising intrinsic mechanisms that detect and protect against infection. Central to this process are pattern recognition receptors (PRRs), which sense pathogen-associated molecular patterns (PAMPs) and danger-associated molecular patterns (DAMPs), thereby initiating an immune response. In addition, the induction of a systemic innate immune response also relies on collaboration between different cell types. Ultimately, these cellular mechanisms are employed to capture and eliminate invading pathogens or to recruit additional innate or adaptive immune cells to affected sites (17). However, aberrant activation of these cells and signaling pathways may consequently lead to an autoimmune attack (14, 27).

**Figure 1:**
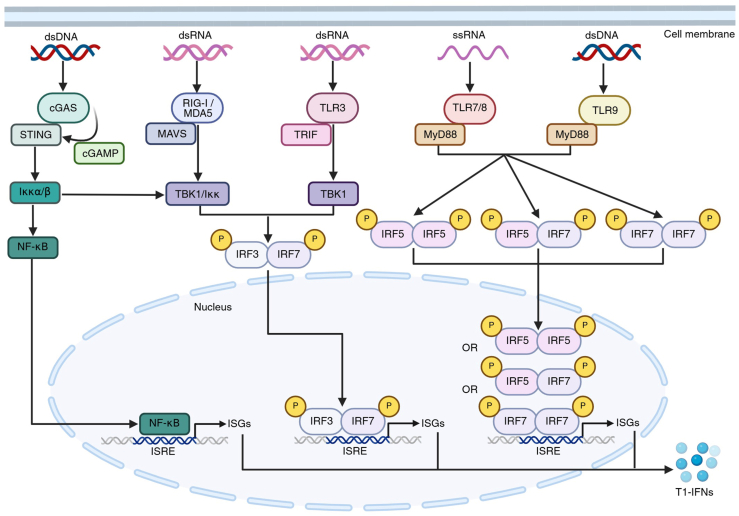
**Schematic overview of the nucleic acid sensor signaling cascades.** cGAS undergoes conformational changes upon dsDNA binding, activating its catalytic function. The second messenger cyclic guanosine monophosphate–adenosine monophosphate (cGAMP) is generated to bind and activate the adaptor protein STING, which then activates two distinct downstream molecule complexes. The TANK binding kinase 1 (TBK1)/inducible IκB kinase (Iκκ) complex induces IRF3–IRF7 heterodimerization, and the Iκκα–β complex activates NF-κb. When RIG-I and MDA5 bind dsRNA, their caspase recruitment domain (CARD) becomes exposed and interacts with mitochondrial antiviral-signaling protein (MAVS [also known as IFNβ promoter stimulator-1 (IPS-1)]). MAVS activates TBK1 and Iκκ, which phosphorylate and activate IRF3 and IRF7. Among the TLRs, TLR3 recognizes dsRNA, TLR7 and TLR8 engage ssRNA, and TLR9 recognizes dsDNA. Activated TLR3 recruits its adaptor protein TRIF, which activates TBK1. In turn, TBK1 phosphorylates IRF3 and IRF7, causing their heterodimerization. Ligand binding by TLR7, TLR8, or TLR9 induces MyD88-mediated IRF5 and/or IRF7 phosphorylation, which subsequently form homodimers or heterodimers. The IRF homodimers and heterodimers and NF-κb translocate to the nucleus and bind to interferon-sensitive response elements (ISREs) to induce transcription of their target ISGs, including T1-IFNs. Figure created with BioRender. cGAS, cyclic GMP–AMP synthase; IRF, interferon regulatory factor; ISG, interferon-stimulated gene; MAVS, mitochondrial antiviral signaling protein; MDA5, melanoma differentiation–associated protein 5; RIG-I, retinoic acid–inducible gene I; STING, stimulator of interferon genes; T1-IFN, type I interferon; TLR, Toll-like receptor.

PAMPs consist of exogenous products derived from microbes, including viral RNA or bacterial lipopolysaccharide. In contrast, DAMPs are endogenous signals derived from stressed, damaged, or dying cells, such as heat-shock protein, mitochondrial DNA, and RNA (28, 29). Upon activation following ligand binding, PRRs recruit adaptor proteins that ultimately lead to the transcription of cytokines, particularly T1-IFNs like IFN-α and IFN-β (Fig. 1). While PRRs bind diverse ligands, the signaling cascades converge on NF-κB or interferon-regulatory factor (IRF) homodimers and heterodimers, which translocate to the nucleus to function as transcription factors and stimulate T1-IFN production (11, 12, 30) (Fig. 1). This promotes inflammation and, in some cases, cell death to eliminate infected or damaged cells (11, 12). However, PRR recognition of endogenous DNA or RNA under conditions of cellular stress or damage might drive an aberrant activation that is often associated with excessive immune activation and autoimmunity (27, 31). This process is predominantly mediated by T1-IFNs (30, 32).

Secreted T1-IFNs bind to IFN-α receptors 1 and 2 on adjacent, uninfected cells and stimulate the transcription of ISGs and human leukocyte antigen in a paracrine fashion, thereby enhancing the proinflammatory response (11, 12, 15). In addition, T1-IFNs act on the IFN-producing cell in an autocrine manner, reinforcing the proinflammatory state. T1-IFNs further boost this response by enhancing the cytotoxic activity of NK cells and increasing the expression of immune effector molecules perforin 1 and granzyme B in cytotoxic T lymphocytes (11, 24, 33). Due to these proinflammatory effects and the connection between innate and adaptive immunity, insight into PRR-driven T1-IFN responses is essential for understanding innate immune dysregulation in autoimmunity.

### Dysregulation of innate immunity mechanisms

Many genetic and epigenetic factors affect key immune regulatory pathways, including mutations in PRRs, which influence their downstream signaling cascades (3, 15, 34). For instance, mutations in *MDA5* (melanoma differentiation–associated protein 5) and consequent aberrant T1-IFN production have been detected in several autoimmune diseases, including SLE and T1DM (27). Other components of innate immunity are also associated with genetic predisposition to autoimmune diseases. Overexpression of proinflammatory cytokines is related to certain autoimmune disorders, as is the case with tumor necrosis factor (TNF) in polyarthritis (14). Epigenetic reprogramming, including DNA hypomethylation and histone modifications, can also contribute to autoimmunity, like in the *IRF7* gene in SLE patients, which causes persistent T1-IFN signaling and immune activation upon its hypomethylation across multiple innate immune cells (30). Besides TNF and T1-IFNs, a wide range of cytokines is involved in innate immune responses and can contribute to autoimmune disease pathogenesis (17). However, especially the hyperactivation of T1-IFN responses plays a key role in multiple autoimmune diseases, including SLE and dermatomyositis (30, 32); Baechler *et al.* (35) reported an increased expression of IFN-regulated genes in around 50% of SLE patients, forming a distinct T1-IFN signature correlating to disease severity. Endogenous DAMPs can also elicit inflammation in autoimmunity in a process known as “sterile inflammation” (34, 36, 37, 38). For instance, mitochondrial DNA that leaks into the cytosol because of mitochondrial dysfunction can activate the cyclic GMP–AMP synthase–stimulator of interferon genes pathway, driving inflammation even in the absence of infection (28, 32, 39). Moreover, chronic DAMP release because of cellular stress or damage can perpetuate low-grade inflammation and contribute to autoimmunity by sustaining activation of immune and inflammatory pathways, as observed in mouse models of Aicardi–Goutières syndrome and ataxia telangiectasia (15, 40, 41, 42).

Activation of specific PRR pathways can trigger signaling cascades that result in transcriptional, metabolic, and epigenetic changes to enhance host defense in a process known as trained immunity, which can lead to chronic inflammation, a hallmark of many autoimmune diseases (15, 29, 37, 38). For example, in a systemic sclerosis mouse model induced by hypochlorous acid, exposure to the Bacillus Calmette–Guérin vaccine was associated with hyperinflammation and exacerbated disease symptoms, including increased fibrosis and skin thickness (43). Similarly, mechanisms that induced trained immunity, such as following a Bacillus Calmette–Guérin vaccination, also induce T1-IFN production. Such “maladaptive trained immunity” is observed in various autoimmune diseases, including SLE, Sjögren’s syndrome, and systemic sclerosis, involving the presence of T1-IFN signatures prior to full disease onset (7, 8, 9, 10). Similarly, another study investigated the effects of beta-glucan administration in a well-established SLE mouse model, which were found to aggravate disease progression (44). These findings underscore a role of trained immunity in autoimmunity. However, while trained immunity is found to enhance disease exacerbation, its involvement in autoimmune disease pathogenesis requires further investigation.

### Activation of the adaptive immune system and autoimmune attack

Although the paradigm-shifting concept suggests that innate immune mechanisms can trigger autoimmune responses, activation of the adaptive immune systems is still required for a full autoimmune response. Crosstalk between the innate and adaptive immune system plays a crucial role in this process, as the innate immune system not only employs multiple mechanisms to regulate infections but also orchestrates the recruitment of adaptive immune cells to boost the immune response (15, 29).

Activation of PRRs on innate immune cells, particularly dendritic cells (DCs), not only recruits and activates innate immune mechanisms but also plays a central role in initiating and shaping adaptive immunity (3, 34, 45). Upon recognition of PAMPs or DAMPs, DCs act as key mediators by producing proinflammatory cytokines and chemokines, such as IL-12 and C-X-C motif chemokine ligand 9, that recruit adaptive immune cells to the site of infection or inflammation (46, 47, 48). Among the recruited immune cells, T cells are crucial for a functional adaptive immune response and interact with DCs to become fully activated (14, 17, 45). In addition, in case of endogenous DAMP recognition, this process can contribute to breaking self-tolerance, which is key to the formation of autoreactive T cells (45, 49, 50). Through these mechanisms, DCs and cytokines not only form the bridge to adaptive immunity but also play a key role in establishing the nature of the adaptive immune response (17, 29). These mechanisms highlight how both the innate and adaptive immune systems work in concert to perpetuate the immune response in autoimmunity.

## Hypoxia and the HIF-1α pathway

Oxygen is essential for the functioning of almost all human cells. Many mechanisms are in place to ensure the homeostatic state of cells when their oxygen supply fails to meet demand (*i.e.*, hypoxia). Under such hypoxic conditions, various downstream pathways are activated to enable cellular adaptation. The key pathway involved in the transcriptional response to hypoxia is the HIF-1α pathway. The HIF family consists of an oxygen-sensitive α subunit (HIF-1α, HIF-2α, and HIF-3α) and a constitutively expressed β subunit (HIF-1β). HIF-1α is expressed ubiquitously, whereas HIF-2α and HIF-3α exhibit more restricted expression patterns. In addition, while HIF-2α and HIF-3α are similarly oxygen sensitive, they have been found to become upregulated less rapidly than HIF-1α under mild or chronic hypoxia (22, 51).

**Figure 2:**
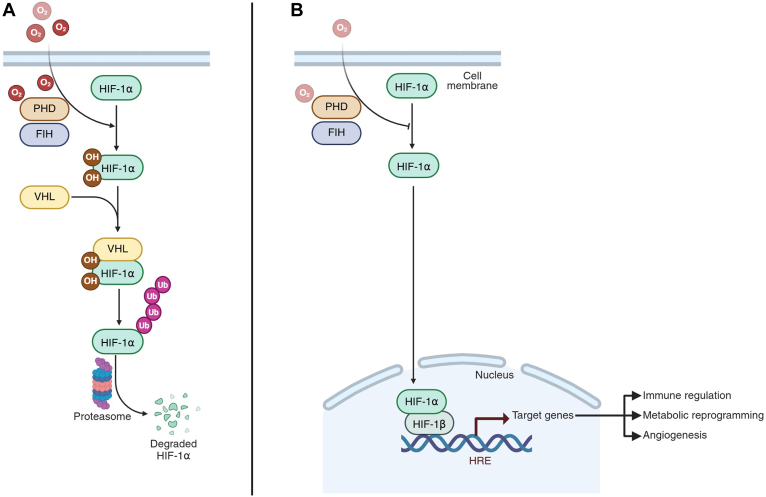
**The HIF-1α pathway in normoxia and hypoxia.***A*, under normoxic conditions, oxygen molecules enter the cell and activate PHD and FIH molecules to hydroxylate proline and asparagine residues on HIF-1α. VHL proteins recognize the hydroxylated HIF-1α molecules and mediate HIF-1α ubiquitination. Subsequently, ubiquitinated HIF-1α molecules undergo proteasomal degradation. *B*, under hypoxic conditions, the insufficiency of oxygen molecules inhibits PHD- and FIH-mediated proline and asparagine hydroxylation of HIF-1α. Activated HIF-1α forms a heterodimer with HIF-1β, which serves as a transcription factor. The HIF-1α–HIF-1β heterodimer binds to hypoxia-response elements on the DNA to induce transcription of target genes involved in various biological processes, including immune regulation, metabolic reprogramming, and angiogenesis. Figure created in BioRender. FIH, factor-inhibiting HIF; HIF-1α, hypoxia-inducible factor-1α; PHD, prolyl hydroxylase; VHL, von Hippel–Lindau.

Under normoxic conditions, in the presence of sufficient oxygen, the HIF-1α pathway is inhibited. Cytosolic O_2_ molecules that are not used in the mitochondria for oxidative phosphorylation mediate HIF-1α hydroxylation by the prolyl hydroxylase (PHD) family and the asparaginyl hydroxylase factor–inhibiting HIF (FIH). PHD-induced hydroxylation allows subsequent ubiquitination of HIF-1α by the von Hippel–Lindau (VHL) tumor suppressor protein, whereas hydroxylation by FIH reduces the transcriptional activity of HIF-1α. The ubiquitinated HIF-1α molecules are marked for proteasomal degradation, preventing activation of the downstream signaling cascade. However, in hypoxic conditions, lack of cytosolic O_2_ prevents PHD and FIH activation, thus inhibiting HIF-1α hydroxylation, ubiquitination, and proteasomal degradation, and facilitating its function as a transcription factor (22, 26). This allows activation of the HIF-1α pathway through nucleus translocation, heterodimerization with HIF-1β, and recruitment of other transcriptional cofactors. The heterodimer functions as a transcription factor and binds to hypoxia-response elements to induce transcription of HIF-target genes. Subsequently, many downstream proteins are upregulated that control cellular adaptation to hypoxic conditions, including immune regulation, metabolic reprogramming, and angiogenesis (23, 25, 26) (Fig. 2).

The direct effects of HIF-1α in immune cells have been described extensively. For instance, HIF-1α commonly induces metabolic reprogramming by causing a shift in metabolism from oxygen-dependent oxidative phosphorylation to glycolysis, which shifts macrophages toward a proinflammatory M1 phenotype (24, 26, 52). This process is described in several autoimmune diseases, including inflammatory bowel disease (IBD) and SLE, where HIF-1α contributes to disease pathogenesis or progression by shaping proinflammatory M1 macrophages (53, 54, 55). In neutrophils, HIF-1α induces NF-κB and reactive oxygen species production to promote their survival and antimicrobial activity by regulating the expression of antimicrobial peptides and the production of proinflammatory cytokines like TNF-α (56, 57). In addition, HIF-1α has been found to enhance the expression of human leukocyte antigen and costimulatory molecules CD80 and CD86 on DCs, which is required for antigen presentation to T cells (58, 59).

However, the mechanisms through which HIF-1α pathway activation affects parenchymal cells, such as β cells and neuronal cells, still require further investigation and evaluation (60, 61). While HIF-1α exerts some generally well-established biological roles, other mechanisms are context and cell specific. Beside metabolic reprogramming, HIF-1α also mediates the shift of lipid metabolism toward the synthesis of lipids and fatty acids (25, 26). HIF-1α is also well known for promoting angiogenesis, most significantly through the upregulation of vascular endothelial growth factor (23, 25, 51). In addition, HIF-1α is involved in cell proliferation, but opposing effects have been documented: HIF-1α signaling has been shown to promote the proliferation of certain cells, such as cancer cells, while inhibiting the proliferation of various other cell types, including hematopoietic stem cells (25, 26, 62). In this context, proliferation is stimulated in cancer cells through mutations in phosphatase and tensin homolog and upregulated vascular endothelial growth factor, among other pathways; however, HIF-1α may inhibit proliferation in many cell types through mechanisms involving p21-induced cell cycle arrest, regulation of proapoptotic factors, such as p53, Nix, and BNIP3, as well as regulation of autophagy-related factors, such as ATG5 (25, 63, 64, 65).

While the role of HIF-1α may be nuanced and context dependent in parenchymal cells, it generally exerts proinflammatory and immunostimulatory effects in immune cells. The differential effects of HIF-1α across various cell and tissue types likely arise from inherent factors, such as tissue-specific oxygen levels. For instance, the oxygen tension in the gastrointestinal tract is lower than in the lungs under normal physiological conditions (23). This disparity in local oxygen availability may influence how HIF-1α regulates cellular functions in distinct tissues (66). These context- and cell-specific roles of HIF-1α suggest that the consequences of HIF-1α accumulation may also differ based on the cellular state. For example, HIF-1α might play a protective role in the physiologically hypoxic intestinal mucosa, while it could exert detrimental effects at the same oxygen tension in the lungs, which experience higher oxygen levels in physiological circumstances. Moreover, hypoxia interacts with other signaling pathways, including HIF-2α, HIF-3α, and NF-κB, further expanding its biological functions (25). The mechanisms through which HIF-1α pathway activation affects parenchymal cells in autoimmune diseases and how this consequently leads to immune suppression or stimulation remain unclear and, in some cases, contradictory (22, 62, 67). In the following sections, we will explore the innate immunomodulatory effects of HIF-1α, focusing particularly on its involvement in the context of autoimmunity.

## HIF-1α pathway and cancer immune suppression

Cancer cells exploit potent immunosuppressive mechanisms that are largely absent in autoimmune diseases, offering valuable insights into how immune regulation might be restored in autoimmunity. Most of the current knowledge on the role of HIF-1α in suppressing immune mechanisms, particularly innate immune pathways, is derived from cancer research. Due to the inability of oxygen to diffuse over distances greater than 200 μm, the oxygen supply to solid tumors fails to meet its demand, rendering the microenvironment hypoxic (24). There, hypoxia acts as an environmental signal that drives tumor cells to adapt to the low-oxygen microenvironment. Tumor cells commonly rely on aerobic glycolysis for ATP generation even in normoxic conditions (Warburg effect). However, the hypoxic microenvironment in solid tumors can further enhance glycolysis through HIF-1α pathway activation, which is associated with tumor progression when HIF-1α is overexpressed (68, 69). This tumor progression is partly mediated by immunosuppressive mechanisms, including HIF-1α-induced shaping of tumor-associated macrophages toward an immunosuppressive and protumorigenic phenotype (70, 71). This finding contrasts with the general HIF-1α-driven proinflammatory M1 phenotype through metabolic reprogramming, supporting the context-dependent role of HIF-1α and suggesting the involvement of additional tumor microenvironmental factors in shaping the macrophage phenotype. The immunosuppressive microenvironment is further enhanced by HIF-1α-induced upregulation of immune checkpoint molecules, cytotoxic T-lymphocyte associated protein 4, programmed cell death protein 1, and its ligand programmed death-ligand 1 on the surface of tumor and immune cells, impairing an appropriate immune response (21).

Importantly, downregulation of the T1-IFN response plays a key role in establishing the immunosuppressive tumor microenvironment (12, 21). While a functional T1-IFN response is crucial for antitumor immunity, downregulation of this response may contribute to tumor progression (33). Miar *et al.* (24) found that the T1-IFN response is downregulated at mRNA and protein levels in both unstimulated, hypoxic cancer cells as well as in hypoxic cancer cells stimulated with a dsRNA mimic. Under hypoxic conditions, the T1-IFN response was reported to be suppressed at every step of the pathway, including the dsRNA sensors, RIG-I (retinoic acid–inducible gene I) and MDA5, their adaptor protein mitochondrial antiviral–signaling protein (or IFNβ promoter stimulator-1), associated transcription factors, and ISGs. The authors described this phenomenon as being only partially dependent on HIF-1α, as expression of factors required for T1-IFN transcription, including RIG-I and various IRFs, was lower in the wildtype human breast cancer cell lines compared with the corresponding HIF-1α knockout cell line. This suggests that, while HIF-1α does exert certain immunosuppressive effects, alternative pathways, such as HIF-2α and HIF-3α, may be required to fully suppress T1-IFN response activity in hypoxic conditions. However, in the same study, downregulation of nucleic acid sensors, RIG-I and MDA5, was regulated by HIF-1α, and heterogeneous effects were reported across the different cell lines used in their experiments, indicating a potential diversity in HIF-1α dependency among cancer cell lines (24). Collectively, these hypoxia- and HIF-1α-induced immunosuppressive mechanisms facilitate immune escape by tumor cells, a process that could similarly benefit parenchymal cells in the context of autoimmunity. However, research into the specific oxygen-sensitive mechanisms that lead to T1-IFN downregulation is essential to ensure that the appropriate pathway is targeted. Furthermore, while this review focuses on HIF-1α, other hypoxia-responsive transcriptional regulators, including HIF-2α, have been implicated in inflammatory and immune signaling in a cell type- and context-dependent manner and are discussed elsewhere (52, 72, 73).

## Hypoxia and the HIF-1α pathway in autoimmunity

### HIF-1α pathway and immune suppression in autoimmunity

While a double-edged sword typically has two sharp sides, we could also regard the suppressive effects of HIF-1α on innate immune mechanisms as a blunt edge. Even though many studies have focused on HIF-1α as wielder of the sharp edge in autoimmunity, research on IBD, T1DM, multiple sclerosis (MS), and psoriasis has uncovered immunosuppressive mechanisms (Table 1) like those that HIF-1α exerts in cancer.

**Table 1:** Overview of the immunosuppressive and immunostimulatory effects of HIF-1α in autoimmune diseases

Autoimmune disease	Immunosuppressive effects of HIF-1α	Immunostimulatory effects of HIF-1α
IBD	HIF-1α expression or stabilization correlates with an enhanced intestinal epithelial barrier function and reduced inflammation (75, 76, 78) PHD inhibitor–induced HIF-1α stabilization reduces mucosal inflammation in a murine colitis model (77)	HIF-1α–induced metabolic reprogramming to glycolysis drives human macrophages toward a proinflammatory M1 phenotype (54)
T1DM	HIF-1α deletion increases susceptibility to streptozotocin- and coxsackievirus-triggered T1DM in mice (82, 83) Activation of the HIF-1α–PFKFB3 pathway protects a rat insulinoma cell line from cytokine-induced apoptosis (130)	N/A
MS	DMF inhibits proinflammatory cytokines like TNF-α in murine splenocytes (84, 85, 89) In a murine experimental autoimmune encephalomyelitis model, HIF-induced EPO expression in neuronal cells protects from neuroinflammation and may counteract BBB permeability (88)	IL-1β–induced HIF-1α activation increases BBB permeability and facilitates immune cell infiltration in primary human fetal astrocyte cultures and rat brains (90)
SLE	N/A	IgG immune complex–induced HIF-1α activation promotes glycolysis, driving a shift toward a proinflammatory M1 macrophage phenotype and the consequent production of proinflammatory cytokines, including IL-1β, in lupus-prone mice as well as in human and murine macrophages (55) HIF-1α silencing decreases IL-17 production and immune complex deposition (IgG and C3) in lupus-prone mice (91)
RA	N/A	HIF-1α induces proinflammatory cytokine production, including IL-1, IL-6, IL-8, and TNF-α, in patient-derived RASFs (93, 94) The signal transducer and activator of transcription 3–HIF-1α–fascin-1 axis mediates RASF migration and invasion through collagen by increasing MMP expression (97, 98) HIF-1α knockdown in human FLSs reduces citrullinated peptide production and PAD2 upregulation, which is involved in peptide citrullination (95)
Psoriasis	FAEs, including DMF, reduce inflammation in psoriasis through inhibition of PHD and consequent activation of HIF-1α (85, 86, 87, 89)	Increased HIF-1α expression in psoriatic lesions is associated with increased production of proinflammatory cytokines, like IL-6 (99, 100)
Dermatomyositis	N/A	HIF-1α stimulates T1-IFN production in the skeletal muscle cells of dermatomyositis patients (106)

N/A, not available; RASF, RA synovial fibroblast.

#### HIF-1α pathway and immune suppression in IBD

Research on IBD suggests that activation of HIF-1α may be protective against innate immune processes in the initiation and progression of autoimmunity. Notably, decreased HIF-1α expression correlates with more severe colitis in mice, whereas increased expression correlates with reduced intestinal epithelial barrier damage and inflammation, which are key features of IBD, in both mice and patient biopsies (74, 75, 76, 77, 78). In addition, Karhausen *et al.* and others (75, 78, 79, 80) have reported that hypoxia is increased at inflamed sites of the intestinal mucosa, where HIF-1α activation is likely a protective mechanism to induce wound healing and recovery of barrier function. Although this immunosuppressive and cytoprotective role of HIF-1α was reported in mice with established colitis, Cummins *et al.* (77) described intestinal epithelial barrier impairment as an early process in IBD pathogenesis, suggesting that HIF-1α may exert protective effects not only during symptom resolution but also prior to full disease onset. Recently, Jacobs *et al.* (81) presented evidence of the effect of hypoxia on innate immunity regulation of intestinal epithelial cells against viral infections, showing that hypoxia and HIF-1α activation limit inflammation against commensal microbes through protein phosphatase 2A activation but may also heighten susceptibility to enteric pathogens. Given the physiological hypoxic nature of the intestinal epithelium, HIF-1α activation may exert protective effects by maintaining epithelial barrier integrity, supporting an immunosuppressive role of HIF-1α in autoimmunity.

#### HIF-1α pathway and immune suppression in T1DM

Studies in mice reported a causal effect between HIF-1α deletion in mouse β cells and T1DM development following treatment with multiple low doses of streptozotocin, a well-established model for inducing T1DM through selective destruction of β cells (82, 83). While this β-cell destruction is not immune mediated and thus does not fully represent an autoimmune response, Lalwani *et al.* (83) additionally reported an increased susceptibility to coxsackievirus-triggered T1DM in mice upon β-cell–specific HIF-1α deletion, suggesting that HIF-1α influences immunomodulation early in T1DM pathogenesis. These findings propose a dual role of HIF-1α in regulating the T1-IFN response. While not explicitly mentioned, the viral clearance in HIF-1α-expressing β cells most likely relies on a functional T1-IFN response, which Lalwani *et al.* underscore by their finding that MDA5 expression was decreased upon HIF-1α knockdown. However, mice with β-cell–specific HIF-1α deletion exhibited a stronger T1-IFN response following viral infection, resulting in T1DM development (83). These findings suggest that HIF-1α may limit T1-IFN response to prevent autoimmunity. Arnaiz *et al.* (12) supported this notion by suggesting that an acute T1-IFN response is crucial for antiviral immunity but that aberrant signaling could contribute to tissue injury. Thus, while HIF-1α boosts the T1-IFN response to inhibit viral replication, it may negatively regulate T1-IFN signaling to prevent cell death and autoimmunity. These findings underscore the role of HIF-1α as a context-dependent modulator of innate immunity, functioning as a “metabolic checkpoint” for T1-IFN signaling.

#### HIF-1α pathway and immune suppression in MS

Similar to the context-dependent inhibitory effects of HIF-1α on T1-IFN production, a study by Albrecht *et al.* (84) on MS reported that administration of the fumaric acid ester (FAE) dimethyl fumarate (DMF) inhibited production of proinflammatory cytokines like TNF-α in murine splenocytes and offered neuroprotection, indicating a protective role of DMF in peripheral immunomodulation. While this study does not focus on the involvement of HIF-1α, the mechanisms of action of FAEs include inhibition of PHD, which in turn activates many downstream targets, such as HIF-1α (85, 86, 87). Therefore, HIF-1α activation could potentially play a role in FAE-mediated immunomodulation. Moreover, in a murine experimental autoimmune encephalomyelitis model, HIF-induced erythropoietin (EPO) expression in neuronal cells protected from neuroinflammation, counteracting blood–brain barrier (BBB) permeability and supporting the potential immunosuppressive role of HIF-1α in MS (88).

#### HIF-1α pathway and immune suppression in psoriasis

Certain FAEs, including DMF, have already been implemented in the treatment of psoriasis and relapsing–remitting MS for more than 5 decades (86, 89). Similar to their effects in MS, FAEs, such as DMF, reduce inflammation in psoriasis, which may be achieved through inhibition of PHD and consequent activation of HIF-1α, among other pathways (85, 86, 87, 89). Thus, FAE-induced HIF-1α activation may lead to suppression of the innate immune system and thereby contribute to prevention of autoimmunity. However, further experimental and clinical studies are necessary to validate this hypothesis and clarify the underlying mechanisms.

Together, these findings suggest that HIF-1α exerts innate immunosuppressive effects in autoimmunity (Table 1). However, the role of HIF-1α in immunomodulation remains multifaceted and context dependent. Therefore, the following section will explore the potential of HIF-1α to drive innate immune responses in autoimmunity.

### HIF-1α pathway and immune stimulation in autoimmunity

In contrary to its blunt, immunosuppressive edge, evidence suggests that hypoxia-induced HIF-1α pathway activation can also act as the sharp edge and stimulate innate immune mechanisms. Hypoxia is a common pathophysiologic feature at sites of inflammation, such as in autoimmune lesions of the skin and vessels in psoriasis and MS, in which hypoxia promotes inflammation (23, 26). This is supported by the overexpression of HIF-1α in such hypoxic autoimmune lesions, where it drives cellular metabolic reprogramming in contrast to the wound healing and barrier protective effects reported previously in IBD (22, 26, 67). Furthermore, HIF-1α contributes to innate immune stimulation through hypoxia-induced mitochondrial damage in tumor and other cells (12). As a consequence, mitochondria-derived DAMPs may be released, which trigger a T1-IFN response and thereby activate the innate immune system (12, 30, 32). While cancer cells often employ immunosuppressive mechanisms to evade an immune response, such as the downregulation of the cyclic GMP–AMP synthase–stimulator of interferon genes pathway or HIF-1α-induced RIG-I and MDA5 downregulation, autoimmune diseases may lack such strategies (12). Moreover, contrary to the immunosuppressive mechanisms outlined above, activation of the hypoxia-induced HIF-1α pathway may instead stimulate the production of proinflammatory cytokines. This contrasts with the hypothesis that HIF-1α negatively regulates T1-IFN signaling to prevent autoimmunity. While HIF-1α may serve as a metabolic checkpoint for T1-IFN signaling, the context-dependent regulatory function of HIF-1α may become aberrant and contribute to dysregulated hypoxia–induced innate immunomodulation.

#### HIF-1α pathway and immune stimulation in IBD

Contrary to the described immunosuppressive effects of HIF-1α activation in IBD, another study found that HIF-1α-induced metabolic reprogramming to glycolysis drives human macrophages toward a proinflammatory M1 phenotype (54). Given the high prevalence of such macrophage polarization induced by HIF-1α metabolic reprogramming, this finding supports the potential immunostimulatory role of HIF-1α in IBD.

#### HIF-1α pathway and immune stimulation in MS

Contrary to the previously described innate immunosuppressive effects, IL-1β-induced activation of HIF-1α was shown to increase BBB permeability in primary human fetal astrocyte cultures and in rat brains, thereby facilitating immune cell infiltration (90). Disruption of the BBB is an early and critical event in the development of MS lesions. However, it remains unclear whether HIF-1α also contributes to systemic immune priming. Moreover, in this study, HIF-1α activation was induced by IL-1β, indicating that the innate immune system was already activated. Therefore, HIF-1α may not represent the primary driver of the observed immune cell infiltration but rather act downstream of existing inflammatory signaling.

#### HIF-1α pathway and immune stimulation in SLE

In keratinocyte-specific HIF-1α knockout mice, the observed decrease in inflammatory cell infiltration of the skin underscores the involvement of HIF-1α in immune cell recruitment (56). In addition, HIF-1α silencing through shRNA transfection prior to full disease onset in a lupus-prone mouse model caused a decrease in IL-17 production and immune complex deposition in lupus-prone mice (91). IL-17 signaling is a key player in the development of fatal glomerulonephritis in lupus (55, 92). Moreover, in both lupus-prone mice and human and murine macrophage cell lines, IgG immune complex–induced HIF-1α activation promoted glycolysis, driving a shift toward a proinflammatory M1 phenotype and resulting in the production of proinflammatory cytokines like IL-1β (55). These findings underscore the innate immunostimulatory role of HIF-1α activation in SLE.

#### HIF-1α pathway and immune stimulation in rheumatoid arthritis

Like findings in SLE, activation of HIF-1α under hypoxic conditions or through plasmid-mediated overexpression was shown to increase the production of proinflammatory cytokines, including IL-6 and IL-8, in patient-derived rheumatoid arthritis (RA) fibroblast-like synoviocytes. This cytokine upregulation promoted synovial inflammation and enhanced immune cell interactions, key features of RA that contribute to joint damage (93, 94). Conversely, HIF-1α knockdown using siRNA reduced proinflammatory cytokine production. While these results were obtained from material derived from patients with established disease, HIF-1α siRNA-mediated knockdown in human fibroblast-like synoviocytes reduced the production of citrullinated proteins. These proteins are key targets of anticitrullinated protein antibodies, which play a central role in RA pathogenesis. In addition, HIF-1α knockdown reduced the upregulation of PAD2, an enzyme involved in peptide citrullination (95). The involvement of HIF-1α in protein citrullination, together with the presence of anticitrullinated protein antibodies years before clinical onset, suggests that HIF-1α may contribute to early disease pathogenesis (95, 96). Moreover, the signal transducer and activator of transcription 3–HIF-1α–fascin-1 axis was found to increase matrix metalloproteinase expression, thereby mediating RA synovial fibroblast migration and invasion through collagen and contributing to disease progression (97, 98).

#### HIF-1α pathway and immune stimulation in psoriasis

Contrary to the described innate immunosuppressive potential of HIF-1α in psoriasis, studies have identified increased HIF-1α levels in skin lesions and serum of psoriasis patients, which were associated with the presence of proinflammatory cytokines, particularly IL-6, thereby contributing to chronic inflammation (99, 100). However, the source of IL-6 was not specified, indicating that this could potentially be an immune cell–intrinsic effect rather than the result of HIF-1α activation in keratinocytes. Moreover, this finding was obtained in patients with established psoriasis and therefore does not directly reflect an immunostimulatory role of HIF-1α in disease development. Nevertheless, deletion of HIF-1α in keratinocyte cell lines and mouse keratinocytes was reported to increase susceptibility to infection because of reduced antimicrobial peptide expression, such as IL-8, underscoring the importance of HIF-1α in establishing innate immune responses (56, 101). Furthermore, the skin, particularly the epidermis, exists under physiologically low oxygen tension because of limited oxygen diffusion from the dermis, and HIF-1α activity contributes to cell proliferation, barrier formation, and wound healing responses (102, 103, 104, 105). However, studies have shown hyperinflammatory responses upon VHL deletion in psoriasis, supporting also a possible proinflammatory role for HIF-1α activation (57). Since VHL regulates many substrates besides HIF-1α, the precise contribution of the HIF-1α pathway remains complex. Validation using an HIF-1α knockout model could confirm that HIF-1α is responsible for the observed hyperinflammation.

#### HIF-1α pathway and immune stimulation in dermatomyositis

Directly opposing the downregulation of RIG-I and the T1-IFN response by HIF-1α in cancer, HIF-1α-induced T1-IFN production has been linked to RIG-I upregulation in the skeletal muscle cells of dermatomyositis patients (106). While this process was reported in established disease, HIF-1α was also found to increase RIG-I expression in human embryonic kidney cells and human myotubes under hypoxic conditions, emphasizing a potential role of HIF-1α in innate immune stimulation.

**Figure 3:**
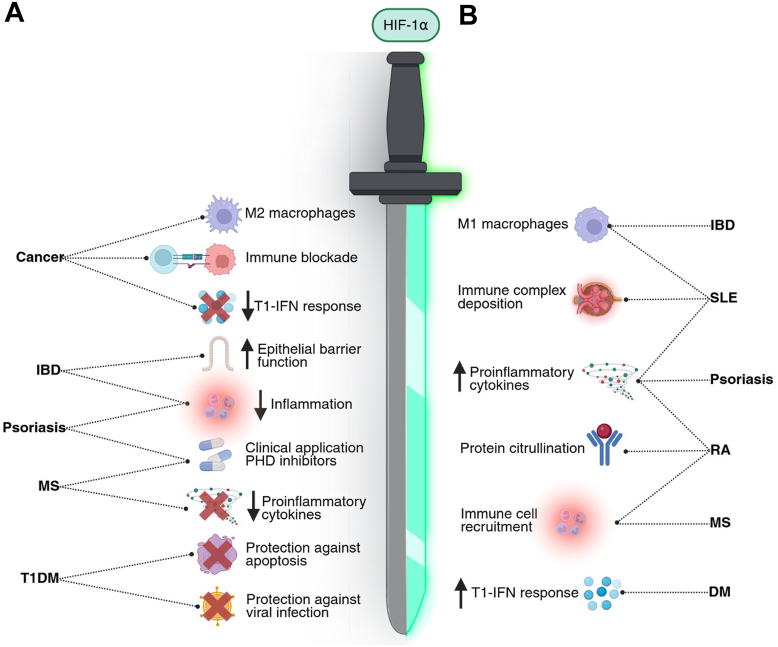
**The immunosuppressive and immunostimulatory effects of HIF-1α.***A*, the blunt edge of the sword represents the immunosuppressive effects of HIF-1α in cancer and across several autoimmune diseases. In cancer, HIF-1α suppresses the innate immune system by inducing the polarization of M2 macrophages, upregulating immune checkpoint molecules, and downregulating the T1-IFN response at several steps, including the downregulation of RIG-I and MDA5. The production of proinflammatory cytokines is inhibited by HIF-1α in MS. In psoriasis and IBD, HIF-1α is involved in resolving inflammation, while also contributing to the function of the intestinal epithelial barrier in IBD. In T1DM, cells are protected from apoptosis and viral infection by HIF-1α activation. *B*, on the sharp edge of the sword, HIF-1α exerts its immunostimulatory effects. In dermatomyositis, HIF-1α upregulates the T1-IFN response through upregulation of RIG-I. Macrophages are polarized to an M1 state by HIF-1α in IBD and SLE. In SLE, HIF-1α also contributes to immune complex deposition and the production of proinflammatory cytokines, a mechanism that is additionally shared by psoriasis and RA. HIF-1α-induced protein citrullination in RA contributes to autoimmune destruction of cells. In addition, HIF-1α contributes to immune cell recruitment in RA and MS. Figure created in BioRender. DM, dermatomyositis; HIF-1α, hypoxia-inducible factor-1α; IBD, inflammatory bowel disease; MDA5, melanoma differentiation–associated protein 5; MS, multiple sclerosis; RA, rheumatoid arthritis; RIG-I, retinoic acid–inducible gene I; SLE, systemic lupus erythematosus; T1DM, type 1 diabetes mellitus; T1-IFN, type 1 interferon.

Contrasting with the previously described immunosuppressive effects of HIF-1α, research on other autoimmune disorders has reported an immunostimulatory role. Besides the upregulation of RIG-I expression and T1-IFN signaling, the production of additional proinflammatory cytokines was associated with HIF-1α expression (Table 1 and Fig. 3). Taken together, these differential effects underscore the role of HIF-1α as a double-edged sword and context-dependent modulator of innate immunity, highlighting the need for further research to underscore which factors determine the edge of the sword HIF-1α wields.

### Pharmacological modulation of HIF-1α in autoimmune diseases

Given the extensive evidence for the role of HIF-1α in the context of inflammation, many studies have suggested the pharmacological potential of HIF-1α modulation. In the context of IBD, HIF-1α stabilization with PHD inhibitors may contribute to maintaining epithelial barrier integrity and suppressing mucosal inflammation (77, 107, 108). Notably, HIF-1α is not the sole target of PHD inhibitors, whose mechanism of action involves the regulation of multiple downstream targets, including NF-κB, HIF-2α, and HIF-3α (109, 110, 111).

Although studies agree that HIF-1α activation protects against environmentally and toxically triggered T1DM in mice, its dual role in wielding both edges of the sword raises the question of which factors contribute to stimulating the T1-IFN response to inhibit viral replication while simultaneously suppressing T1-IFN pathways to prevent autoimmune-mediated β-cell death (82, 83). Underscoring the context dependence, we have recently discussed in a literature review that the effects of HIF-1α could vary depending on the state of β cells (60). While activation of the HIF-1α pathway appeared deleterious in healthy β cells, HIF-1α exerted protective effects in disease-relevant contexts, including metabolic stress, viral infection, and inflammation, consistent with HIF-1α–target gene upregulation observed in islets from individuals with pre-T1DM, as recently reviewed (60). This supports the notion that pharmacological HIF-1α activation in stressed β cells could hold potential to modulate innate immune mechanisms prior to T1DM development. However, further research is required before therapeutical modulation is viable. Beyond determining the conditions in which the edge of the sword shifts from blunt to sharp, studies applying other models of T1DM are required. While viral infections, such as coxsackievirus, are established models for inducing autoimmune diabetes in animals, a direct causal role of viral infection in the onset of T1DM in humans has not yet been demonstrated (112, 113, 114, 115, 116).

The involvement of HIF-1α in MS and psoriasis remains more contradictory, with studies describing not only predominantly immunosuppressive effects but also some immunostimulatory effects. However, FAEs, such as DMF, have been used clinically for the treatment of psoriasis in Europe for over 5 decades and have more recently been approved for relapsing–remitting MS patients (86, 89). As one of their reported mechanisms of action involves PHD inhibition, which can induce HIF-1α activation, the success of FAEs in these autoimmune diseases supports the potential protective effects of HIF-1α stabilization (85). PHD inhibitors were also found to offer neuroprotection and reduce inflammation by suppressing proinflammatory cytokine production in an MS model (84). In addition, the clinically approved PHD inhibitor roxadustat has been shown to increase EPO production in chronic kidney disease–associated anemia (117, 118, 119). Given the well-established benefit of EPO in MS models (88), PHD inhibitors like roxadustat might benefit at-risk MS individuals; however, further investigation is still required to test the effects of PHD inhibitors on EPO production and their safety in MS. Moreover, FAEs employ various mechanisms of action, which do not necessarily rely on PHD inhibition but involve activation of other players, such as HCAR2, and the transcription factor, NRF2 (85, 86, 89, 120). Similarly, the hypoxia-induced immunosuppression in cancer being only partially dependent on HIF-1α highlights the important role of such additional key players (24). However, evidence from Gao *et al.* (121) supports the immunosuppressive role of fumarate-induced HIF-1α in cancer, where fumarate functions as an oncometabolite and activates HIF-1α through fumarate-mediated succination. Further investigation is crucial to fully uncover the specific immunomodulatory mechanisms induced by PHD inhibitors, FAEs, and hypoxia.

Research on SLE and RA has reached a consensus that HIF-1α promotes disease by activating innate immune mechanisms (91, 93, 94), where HIF-1α inhibition, rather than activation, would be protective in these disorders (24, 30, 32, 106). Similarly, HIF-1α inhibition could decrease the production of proinflammatory cytokines, including IL-6 and IL-8, in psoriasis and RA (93, 94, 99, 100). The effectiveness of the HIF-1α inhibitor PX-478 is already established by its suppression of macrophage glycolysis and consequent proinflammatory M1 phenotype, which was additionally associated with decreased immune complex deposition in SLE (122).

Despite the described preclinical evidence, clinical studies are still essential to identify individuals who may benefit from HIF-1α modulation and clarify the context-dependent role of HIF-1α in autoimmunity by taking both edges of the sword into account. Activation of HIF-1α could potentially benefit at-risk IBD, T1DM, MS, and psoriasis patients. Several PHD inhibitors, including roxadustat, have already been approved for clinical use in chronic kidney disease–associated anemia (118). While increased incidence rates of infection have been previously described, clinical evidence does not support an increased incidence of autoimmunity or T1-IFN response in patients treated with PHD inhibitors (123, 124). Moreover, in two different meta-analyses evaluating the safety of roxadustat in patients with chronic kidney disease–related anemia, no drug-related severe adverse effects of any kind were found (125, 126). However, close monitoring of treated patients and further research is needed to fully understand the role of HIF-1α in immune modulation. Thus, clinical trials could be adapted because of the already established efficacy and safety outcomes.

Importantly, integrating data from genetic HIF-1α knockout models *versus* HIF-1α stabilization approaches remains a challenge. Complete knockout abolishes protein function, whereas stabilization (*e.g.*, pharmacological or hypoxic) primarily prevents degradation and may preserve regulatory mechanisms such as FIH-mediated inhibition while engaging compensatory pathways (127, 128). These conditions are therefore not simple opposites and should be interpreted with caution. In addition, the potential protumorigenic effects of HIF-1α should be taken into account, even though clinical evidence of HIF-1α-activating drugs shows no increased risk of cancer (117, 119). While HIF-1α inhibitors may benefit individuals at risk for SLE or RA, they have not been approved yet for clinical use and require clinical trials to assess their efficacy and safety to ensure selective modulation of HIF-1α at the target tissue (129). Beyond the involvement of HIF-1α in the discussed autoimmune disorders, its wide array of innate immunomodulatory effects could play a key role in the entirety of autoimmune diseases. This way, the aim is to pave the way for better understanding of autoimmune disease phenotypes and identify novel targets.

## Future perspectives and conclusion

In this review, we described both edges of the sword of HIF-1α regarding innate immunomodulation in autoimmunity. Cancer research has set the stage for a breakthrough in the immunomodulatory effects of the microenvironment. While hypoxia-induced HIF-1α activation is widely studied in the context of autoimmunity, the direction of the immunomodulatory effects of HIF-1α remains highly debated. We revealed that this direction depends not only on the context but also on other factors such as the affected tissue and cell type. In addition, we assessed the pharmacological potential of HIF-1α modulation to regulate innate immune mechanisms prior to autoimmune disease onset.

The involvement of additional factors or pathways in hypoxia-induced innate immunomodulation in autoimmunity is underscored by the inherent differences in oxygen levels across tissues (23). The ambiguous role of hypoxia and HIF-1α activation in autoimmune disease pathogenesis can either preserve homeostasis or drive pathogenesis depending on the target tissue. Beyond tissue and cell specificity, we discussed additional contextual factors that shape which edge of the sword HIF-1α wields, including the expression of negative regulators, such as VHL and PHD, but also of HIF-1α itself, which differs across tissues and local oxygen levels (23). This diversity further underscores the importance of additional investigation into the immunomodulatory role of varying oxygen levels and physiological hypoxia in autoimmunity. Taken together, these contradictory findings underscore the need for further insight into the factors dictating the immunomodulatory features of chronic T1-IFN signaling and how HIF-1α balances antiviral immunity and autoimmunity through modulation of the T1-IFN response.

In conclusion, the immunomodulatory role of HIF-1α is highly context dependent, varying across parenchymal cell types, autoimmune diseases, and even with the activation state of the affected cells or the stage of disease progression. These observations highlight the complexity of HIF-1α-mediated signaling in immune regulation and suggest that the effects of HIF-1α may shift between protective and pathogenic functions depending on the inflammatory environment. Consequently, targeted pharmacological modulation of the HIF-1α pathway, through either activation or inhibition, depending on the disease context and tissue involved, may represent promising strategies to prevent or mitigate the development and complications of autoimmune diseases.

## Conflict of interest

The authors declare that they have no conflicts of interest with the contents of this article.
